# A Mutated PB1 Residue 319 Synergizes with the PB2 N265S Mutation of the Live Attenuated Influenza Vaccine to Convey Temperature Sensitivity

**DOI:** 10.3390/v12111246

**Published:** 2020-10-31

**Authors:** Andrew Cox, Jordana Schmierer, Josephine D’Angelo, Andrew Smith, Dustyn Levenson, John Treanor, Baek Kim, Stephen Dewhurst

**Affiliations:** 1 Department of Microbiology and Immunology, University of Rochester School of Medicine and Dentistry, Rochester, New York, NY 14642, USA; andrew.cox@cchmc.org (A.C.); jordana_schmierer@urmc.rochester.edu (J.S.); dangeloj@upstate.edu (J.D.); Andrew_Smith72@urmc.rochester.edu (A.S.); dlevenson@med.wayne.edu (D.L.); John.Treanor@hhs.gov (J.T.); baek.kim@emory.edu (B.K.); 2 Medical Scientist Training Program, University of Rochester School of Medicine and Dentistry, Rochester, New York, NY 14642, USA; 3 Department of Pediatrics, Pediatric Residency Program, Cincinnati Children’s Hospital Medical Center, Cincinnati, OH 45229, USA; 4 Upstate Medical School, State University of New York, Syracuse, NY 13210, USA; 5 M.D./Ph.D. Training Program, Wayne State University, Detroit, MI 48202, USA; 6 Division of Infectious Diseases, Department of Medicine, University of Rochester School of Medicine and Dentistry, Rochester, New York, NY 14642, USA; 7 Biomedical Advanced Research and Development Authority (BARDA)/HHS/ASPR, Influenza and Emerging Diseases Division 21J14, 200 C St SW, Washington, DC 20515, USA; 8 Department of Pediatrics, Emory University, Atlanta, GA 30322, USA; 9 Center for Drug Discovery, Children’s Healthcare of Atlanta, Atlanta, GA 30322, USA

**Keywords:** Influenza A Virus, LAIV, temperature sensitivity, vaccine, polymerase

## Abstract

Current influenza vaccines have modest efficacy. This is especially true for current live attenuated influenza vaccines (LAIV), which have been inferior to the inactivated versions in recent years. Therefore, a new generation of live vaccines may be needed. We previously showed that a mutation at PB1 residue 319 confers enhanced temperature sensitivity and attenuation in an LAIV constructed in the genetic background of the mouse-adapted Influenza A Virus (IAV) strain A/PR/8/34 (PR8). Here, we describe the origin/discovery of this unique mutation and demonstrate that, when combined with the PB2 N265S mutation of LAIV, it conveys an even greater level of temperature sensitivity and attenuation on PR8 than the complete set of attenuating mutations from LAIV. Furthermore, we show that the combined PB1 L319Q and PB2 N265S mutations confer temperature sensitivity on IAV polymerase activity in two different genetic backgrounds, PR8 and A/Cal/04/09. Collectively, these findings show that the PB2 LAIV mutation synergizes with a mutation in PB1 and may have potential utility for improving LAIVs.

## 1. Introduction

Influenza A Viruses (IAV) dramatically impact human health and infect millions of persons per year [1]. Whereas infection is typically self-limited and lasts only a few days, it can cause life-threatening illness in vulnerable populations such as the very young and very old [2]. Between 3000 and 49,000 people die of seasonal influenza each year in the United States, with over 250,000 deaths worldwide. Indeed, 2.8% of global deaths of infants under one year of age and 1.8% of deaths among children aged 1–4 years are attributable to influenza infection [3,4]. Additionally, influenza can undergo major antigenic changes through the process of reassortment, which may lead to devastating and costly pandemics [5,6,7,8,9,10].

Antiviral therapy and vaccination serve as the first line of defense against IAV, but antiviral usage is limited by the increasing prevalence of resistant clinical isolates [11,12,13,14,15,16,17,18,19,20]. Vaccination is therefore the recommended mechanism of protection, and currently, three vaccine strategies are utilized [21,22,23,24,25,26]. The first, inactivated influenza vaccine (IIV), contains either detergent-disrupted virions or purified membrane proteins from the strains projected to circulate in the population the following year [27]. IIV is currently formulated to include two IAV and two influenza B virus strains, which are selected each year by the World Health Organization (WHO) [28,29,30,31,32,33,34,35,36]. The second strategy utilizes purified recombinant IAV hemagglutinin produced in insect cells, which is delivered at a dose three-fold higher than the level of HA present in IIV and also corresponds to the WHO-recommended strains [37,38]. The third option is a live attenuated influenza vaccine (LAIV). The Influenza A LAIV currently licensed in the US consists of six internal gene segments (PB1, PB2, PA, NP, M, and NS) of a cold passaged isolate of A/Ann Arbor/6/60 H2N2, now termed the master donor virus (MDV) [39,40,41,42,43,44,45,46,47,48,49,50,51,52]. HA and NA-the segments, encoding the same surface proteins used in the IIV that year, are added to these six gene segments [26].

The MDV backbone used in seasonal LAIVs was created by passaging A/Ann Arbor/6/60 at decreasing temperatures until growth was achieved at 25 °C [53]. In addition to an increased ability to grow at low temperatures, this strain has decreased viral titers at elevated temperatures with little to no productive replication at temperatures above 37 °C [44]. Further experiments determined that these temperature sensitive (ts) viruses were attenuated in both mice and humans [44]. Temperature sensitivity and attenuation were found to be conveyed by mutations within PB2 (N265S), PB1 (K391E, E581G, A661T), and NP (D34G) [42,45].

Early studies, conducted prior to the availability of contemporary molecular cloning methods, examined the phenotypic stability of wild-type IAVs containing single gene segments from LAIV [43,44,45,46,47,48,49,50,51,52,53,54]. Here, we describe one of these single gene replacement (SGR) viruses, containing the PB2 gene of LAIV in the genetic background of a seasonal H3N2 virus [54]. This SGR virus was found to possess an unexpectedly high degree of temperature sensitivity, which was associated with a novel mutation in PB1 (PB1 319Q). We previously showed that this PB1 mutation confers enhanced temperature sensitivity and attenuation on an LAIV constructed in the genetic background of the IAV strain, A/PR/8/34 (PR8) [55]. Here, we describe the discovery/origin of this mutation and propose a mechanism for its temperature sensitive phenotype.

## 2. Materials and Methods

### 2.1. Cells and Media

Experiments were carried out in HEK 293 FT cells (ATCC), MDCK cells (ATCC), or A549 cells (Sigma). Cells were grown in Dulbecco’s Minimum Essential media (Gibco) supplemented with 10% Fetal Bovine Serum (USA Scientific) and Penicillin/Streptomycin (Gibco) (Growth media). Virus infections were carried out in Dulbecco’s Minimum Essential media (Gibco) supplemented with 0.3% Bovine Serum Albumin Fraction V (Gibco), Penicillin/Streptomycin (Gibco), and 1 µg/mL L-(tosylamido-2-phenyl) ethyl chloromethyl ketone (TPCK) treated Trypsin (Sigma) (Infection media). Minigenome experiments were carried out in Optimem (Gibco) without supplementation. Plaque purifications were performed using warmed overlays of 20% 10X DMEM (Gibco) in ddH20 supplemented with Bovine Serum Albumin Fraction V (Gibco), Glutamax™ (Gibco), gentamycin (Gibco), 5% NaCO3 (Gibco), 1 µg/mL TPCK treated Trypsin (Sigma), and Seakem™ LE (low melting point) Agarose (Lonza) (Purification media).

### 2.2. Plaque Purification

Our initial viral stocks consisted of (i) A/Korea/82 H3N2 HA, and NA with the six internal segments of LAIV; (ii) a SGR with the PB2 segment of LAIV and all other segments from A/Korea/82 (SGR); and (iii) a derivative of this SGR that had been serially passaged at elevated temperatures and had been reported to phenotypically revert (SGR-Rev) [54]. Serial dilutions from 10^−1^ to 10^−6^ of each viral stock were added to confluent six-well dishes of MDCK cells. Cells were incubated at 33 °C for 1 h. Warmed virus purification media was overlaid on the MDCK cells and incubated at 33 °C for 72 h. Ten plaques were chosen from each viral stock and amplified through one round of passage in a confluent T-75 flask at 33 °C. The supernatants of these flasks were harvested 72 h post infection, clarified by centrifugation at 2000 rpm, stored at −80 °C, and titered by TCID_50_ on MDCK cells.

### 2.3. Tissue Culture Infectious Dose for 50% Infection (TCID_50_)

Viral supernatants were diluted from 10^0^–10^−7^ in infection media and used to infect fresh 96-well plates of MDCK cells in quadruplicate for 1 h at 33 °C. Fresh infection media was then added and the plates were incubated at their respective temperatures for 5 days. Upon completion of infection, a hemagglutination assay was performed as described [56,57]. TCID_50_ was calculated by the method of Reed and Muench [58].

### 2.4. Single Reaction Genomic Amplification for IAV Gene Sequencing

IAV vRNA was isolated from the supernatant of infected MDCK cells using the RNA Nucleospin^®^ purification kit (Machery-Nagel), reverse transcribed and amplified using SuperScript™ III One-Step RT-PCR System with Platinum^®^ Taq High Fidelity (Thermo Fisher), and cloned into a modified pHH21 vector as described [59]. The four genes of the ribonucleoprotein (PB1, PB2, PA, and NP) genes were then subcloned into the pCAGGS vector and transformed into XL-1 Blue *E. coli*. When possible, the EcoRI and XhoI sites were used, but due to sequence limitations, some genes required utilization of the XhoI and BglII sites. All genes were sequenced after cloning to confirm that no additional mutations had been added. All plasmids are available upon request.

### 2.5. Minigenome Assay

First, 60% confluent HEK 293FT cells in Poly(ethyleneimine) solution (PEI) (Fisher) coated 24-well plates were transfected with 200 ng pCAGGS-NP, pCAGGS-PA, pCAGGS-PB1, and pCAGGS-PB2; 50 ng pPolI-NP-Luc; and 10 ng pCAGGS-Gaussia in triplicate using in Optimem for 60 min at 37 °C and then transferred to their indicated temperature. Control wells were also transfected with all above plasmids except PB1 (“-PB1 samples”) to control for background luminescence. At 24 h post transfection, cells were lysed using 125 µL Passive Lysis Buffer (Promega) and cleared through centrifugation at 10,000 rpm at 4 °C for 2 min. Next, 25 µL clarified lysates were added to Corning Costar™ white 96-well flat-bottomed optical plates (Fisher). To each well, 25 µL Luciferase Activity Reagent II (Promega) was added, the plate was shaken for 20 s, and a luminescence measurement was made of each well for 2 s using a Beckman Coulter DTX 880 plate reader. Then, 25 µL Stop & Glo^®^ Reagent (Promega) was added to each well, the plate was shaken for 20 s, and a luminescence measurement was made of each well for 2 s using the same Beckman Coulter DTX 880. All reactions were performed in triplicate on at least three separate occasions. The background luminescence detected in the “-PB1 samples” (firefly luminescence divided by Gaussia luminescence) was arbitrarily set as 1. Increases above this were denoted as fold increases in activity. All data were depicted as fold induction, where the firefly luminescence produced by the viral polymerase was normalized to (divided by) the Gaussia luminescence (which is produced by the cells independent of the viral polymerase).

### 2.6. Site-Directed Mutagenesis

Site-directed mutagenesis was performed using the QuikChange II, Lightning, and Lightning Multi Site directed mutagenesis kits (all from Agilent). Two clones possessing the correct mutation were selected from each mutagenesis reaction and used for each minigenome assay to confirm the desired effects.

### 2.7. Viral Rescue

Four different PR8 viruses (wt PR8 (PR8), PR8 containing PB2 N265S (PR8 265S), PR8 containing PB1 L319Q (PR8 319Q), and PR8 containing both PB2 N265S and PB1 L319Q (PR8 265S/319Q)) were created using the bidirectional plasmid system for A/Puerto Rico/8/1934 (H1N1) (a kind gift from Dr. Adolfo García-Sastre, Mt. Sinai School of Medicine) as described [60]. All viruses were plaque purified upon rescue and the polymerase sequenced in its entirety.

### 2.8. Growth Curves

Fresh confluent six-well dishes of A549 cells were infected with virus at a MOI of 0.01. Then, 10% of the supernatant (300 µL) was collected and replaced with fresh media at 12 h, 24 h, 48 h, 72 h, and 96 h post infection. These supernatants were clarified by centrifugation and stored at −80 °C prior to titering by TCID_50_ in MDCK cells.

### 2.9. Viral Attenuation

All experiments were performed as described [61]. First, 5–7-week-old female C57/B6 mice were purchased from Jackson Labs, lightly anesthetized, and infected with increasing doses of virus in 30 µL of infection media. Animals were monitored daily for signs of clinical distress and euthanized at 25% body weight loss. LD_50_ was calculated by the method of Reed and Muench [58]. The data for PR8 319Q were reproduced from our previous work [55].

### 2.10. Ethics Statement

All animal experimentation in this study was reviewed and approved by the University of Rochester’s University Committee on Animal Resources (UCAR). The University of Rochester and its animal research facilities are fully accredited by the Association for Assessment and Accreditation of Laboratory Animal Care, International, and adhere to the humane use of animals, as dictated by the National Institutes of Health’s Office of Laboratory Animal Welfare through the Animal Welfare Act as prescribed by “The Guide for the Care and Use of Laboratory Animals”.

### 2.11. Semi-Infectious Particles

For these experiments, we modified the technique developed by Brooke and Yewdell for measuring semi-infectious particles [62,63]. In brief, MDCK cells were infected at an MOI of 0.01 at 33 °C and 39 °C with (i) PR8 WT, (ii) PB1 319Q, (iii) PB2 265S, (iv) PB1 319Q & PB2 265S, (v) PR8 LAIV (mutations introduced = PB2 N265S, PB1 K391E, PB1 E581G, PB1 A661T), and (vi) PR8 LAIV plus PB1 319Q viruses. After 72 h, viral supernatants were harvested and clarified via centrifugation. Particle levels were then analyzed through hemagglutination assay. The same day of harvest, fresh plates of cells were infected with each of the virus stocks at the permissive temperature of 33 °C using virus concentration of 1 HA unit /10 cells. Viral spread was disrupted by the addition of the HA-neutralizing antibody NR-4542 (kindly provided by the Yewdell lab via BEI) 2 h post infection to ensure a single-cycle infection. At 10 h after antibody addition, cells were gently trypsinized, resuspended, and stained for HA and NA (using antibodies kindly provided by the Yewdell lab via BEI; HA Ab = NR-48783, NA Ab = NR-50239). Then, 250,000 cells were run on a BD Accuri Flow Cytometer, and the ratio of cells positive for HA alone or NA alone was compared to cells expressing both proteins. This experiment was performed in triplicate on three separate dates, each with freshly generated viral stocks.

## 3. Results

### 3.1. Identification of a Single-Gene Replacement Virus That is More Temperature Sensitive than LAIV

We compared the growth kinetics of plaque-purified isolates of three viruses that each contained the PB2 segment of a cold-adapted A/Ann Arbor/6/60 (H2N2) mutant (A/AA/60-LAIV) (which was subsequently used as the basis of the currently licensed LAIV), with the remaining seven segments derived from either this same cold-adapted virus or a seasonal H3N2 strain A/Korea/1982. Specifically, we characterized three related viruses. The first contained the HA and NA gene segments of A/Korea/1982 and the remaining six segments from A/AA/60-LAIV (hereafter referred to as LAIV). The second was a single-gene replacement A/Korea/1982 virus containing only the PB2 segment from A/AA/60-LAIV (hereafter referred to as SGR). The SGR virus was previously shown to retain the ts phenotype of LAIV [54]. The third virus was a derivative of SGR that had been serially passaged at elevated temperatures and had undergone phenotypic reversion of its ts phenotype (hereafter referred to as SGR-Rev [54] (Table 1)).

**Figure 1:**
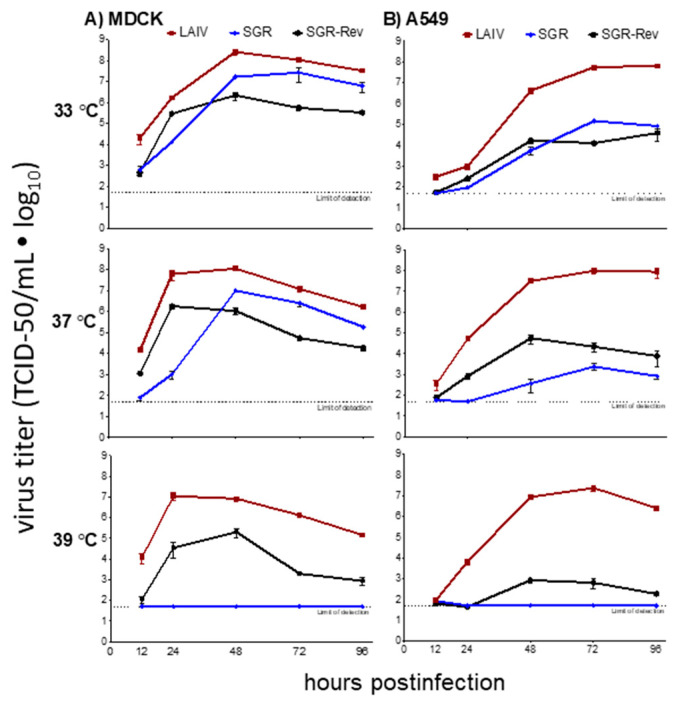
A single gene replacement virus containing the PB2 segment of LAIV serially passaged at 33 °C (SGR) increased temperature sensitivity compared to both LAIV and a revertant derivative of this parental virus (SGR-Rev). Multicycle growth curve experiments were performed at 33 °C, 37 °C, and 39 °C with (**A**) MDCK and (**B**) A549 cells (ATCC) as described [63]. Mean ± SD values for triplicate infections are plotted. The dotted line denotes the limit of detection (50 TCID_50_/mL). All viruses contained the HA and NA genes of A/Korea/82 (H3N2). LAIV denotes a virus containing all six internal segments of the cold-adapted, temperature-sensitive, attenuated (ca ts att) A/AnnArbor/6/60 MDV. SGR and SGR-Rev had the same membrane proteins, but contained only one segment (PB2) from A/AnnArbor/6/60, with all others deriving from a seasonal strain A/Korea/82 H3N2. SGR-Rev represent a derivative of SGR that was passaged at increasing temperatures until growth was again detected at 39 °C [54].

All viruses were plaque purified at 33 °C and 10 plaques of each virus were subjected to a single round of amplification in MDCK cells at 33 °C. The ribonucleoprotein segments (PB1, PB2, PA, and NP) of each viral plaque were then sequenced and found to be identical at the amino acid level across strains in all ten virus clones tested (data not shown). Representative stocks were then analyzed for multicycle growth kinetics in A549 and MDCK cells (Figure 1). LAIV grew at all temperatures (Figure 1). SGR-Rev grew poorly at 39 °C as described (Figure 1), while the parental SGR virus grew at 33 °C and 37 °C, but completely failed to replicate at 39 °C (Figure 1). Therefore, this SGR virus displayed a temperature-sensitive phenotype.

### 3.2. A Single Residue Conveyed the Majority of Phenotypic Reversion

**Figure 2:**
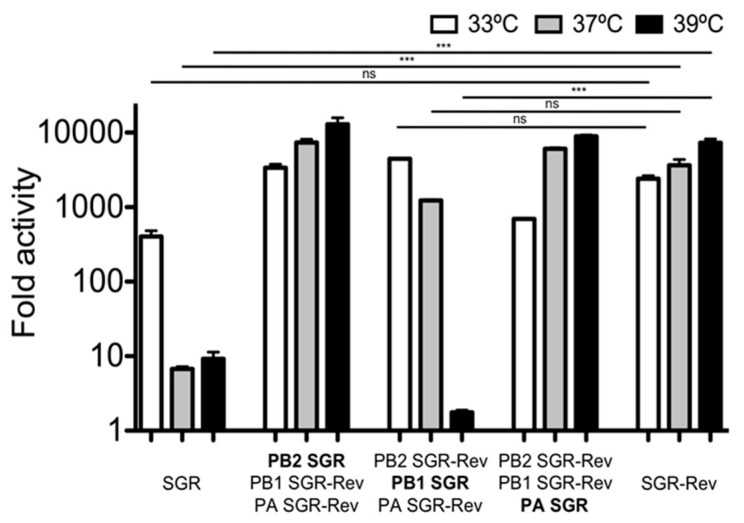
SGR impaired polymerase activity at 37 °C and 39 °C as compared to SGR-Rev, and this was attributable to the PB1 gene segment. Minigenome assays were performed in HEK-293T cells (ATCC) as described [61]. X-axis labels denote the source of the various polymerase components (PB2, PB1, PA) in the minigenome assay. Mean ± standard deviation (SD) fold increase activity over a no-PB1 control is shown. All transfections were performed in triplicate on three separate occasions. Statistics were performed using one-way ANOVA followed by Tukey’s posttest. Ns: Not significant, *** *p* < 0.001.

To determine what mutations were necessary for the temperature sensitivity of the SGR virus, the ribonucleoprotein components of these viruses (PB1, PB2, PA, and NP) were cloned, sequenced, and introduced into the expression vector pCAGGS for further analysis [64]. The PB2 segment was from the A/Ann Arbor/6/60 strain and the PB1, PA, and NP segments from A/Korea/1982. Six amino acid differences were detected between the polymerases of the SGR and SGR-Rev viruses (Table 2). The role of these mutations in imparting temperature sensitivity was then examined using a minigenome assay [57], which revealed temperature sensitivity of the polymerase of the SGR virus at both 37 °C and 39 °C (left panels, Figure 2). In contrast, the polymerase of SGR-Rev displayed robust polymerase activity at both of these temperatures (right panels, Figure 2). Replacement of the PA or PB2 segment from the polymerase of SGR did not impart temperature sensitivity to the polymerase of (SGR-rev) (Figure 2). However, replacement of the PB1 segment of SGR-Rev resulted in temperature sensitive polymerase activity at 39 °C (Figure 2).

**Figure 3:**
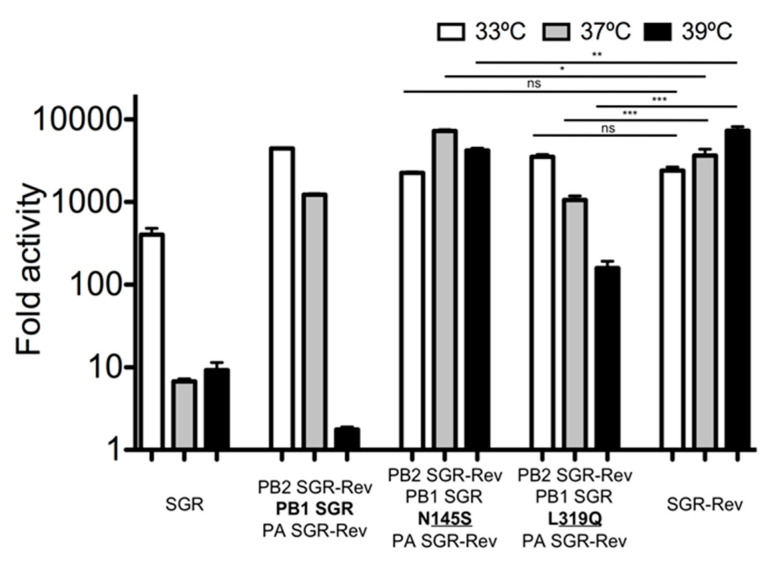
Residue 319Q is primarily responsible for the increased temperature sensitivity of the SGR virus at 37 °C. Minigenome assays were performed in HEK-293T cells (ATCC) as described [61]. X-axis labels denote the source of the various polymerase components (PB2, PB1, PA) in the minigenome assay. Data represent mean ± standard deviation (SD) fold increase activity over a no-PB1 control. All transfections were performed in triplicate on three separate occasions. Data from Figure 2 for groups SGR, PB1 SGR with Rev PB2 and PA, and SGR-Rev were reproduced for comparison. Statistics were performed using one-way ANOVA followed by Tukey’s posttest. Ns: Not significant, * *p* < 0.05, ** *p* <0.01, *** *p* < 0.001.

There are only two amino acid differences between the SGR and SGR-Rev in PB1 (S145N, Q319L; the SGR residue is listed first). We therefore used site-directed mutagenesis (Agilent) to examine the phenotypic effect of these individual mutations-and determined that a mutation from glutamine to leucine at residue 319 was primarily responsible for the increased polymerase activity of the SGR-Rev virus at 39 °C (Figure 3).

**Figure 4:**
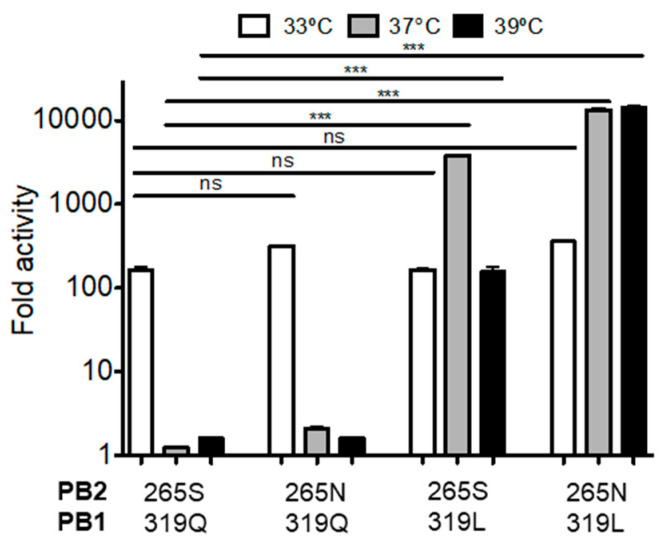
Introduction of the PB2 265N and PB1 319L mutations into the SGR polymerase results in a loss of temperature sensitivity and acquisition of the phenotype of the SGR-Rev virus. Minigenome assays were performed in HEK-293T cells (ATCC) as described [61]. In this experiment, PB2, PB1, and PA were all derived from the SGR virus (PB2 265S, PB1 319Q). Specific site-directed mutations were then introduced and evaluated as indicated in the X-axis label. Data represent mean ± standard deviation (SD) fold increase activity over a no-PB1 control. All transfections were performed in triplicate on three separate occasions. Statistics performed using one-way ANOVA followed by Tukey’s posttest. Ns: Not significant, *** *p* < 0.001.

We next performed a reciprocal experiment, introducing the PB2 265N and PB1 319L mutations into the SGR polymerase (Figure 4). This analysis revealed that the PB1 319 residue was the key driver of the ts phenotype. The introduction of the 319L residue alone was sufficient to mediate loss of temperature sensitivity and acquisition of the phenotype of the SGR-Rev virus (Figure 4).

### 3.3. PB1 319Q and PB2 265S Impart Temperature Sensitivity to the Viral Polymerase of Two Additional IAV Strains

**Figure 5:**
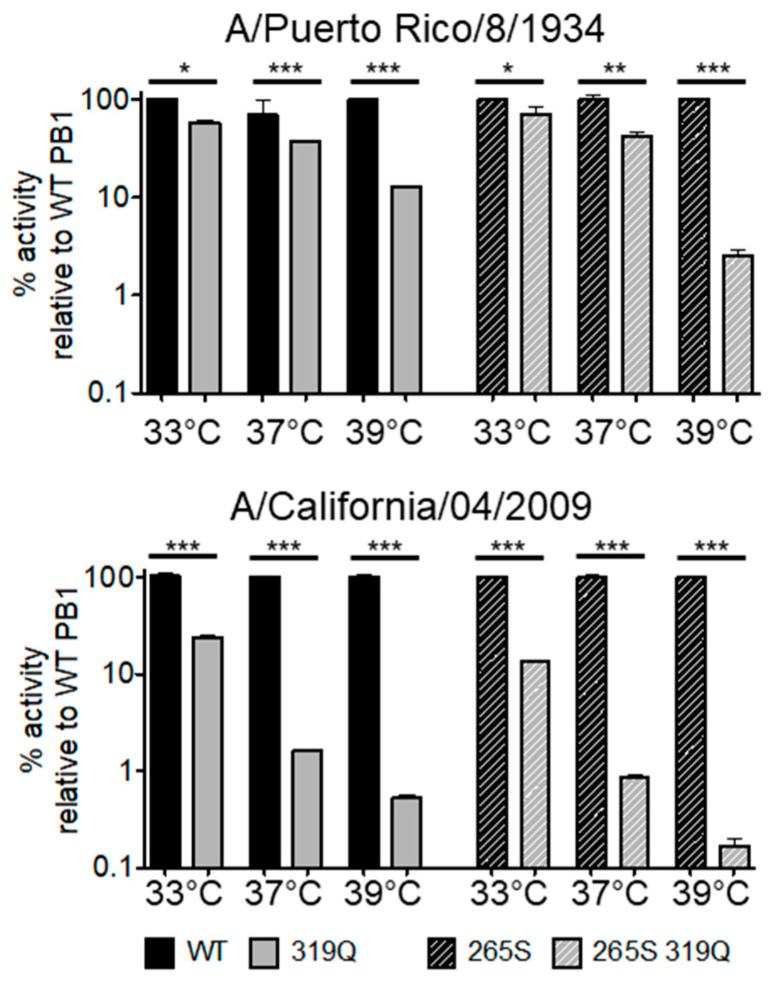
The PB2 265S and 319Q mutations synergistically increased polymerase temperature sensitivity in two disparate polymerases of Influenza A Virus. Minigenome assays were performed in HEK-293T cells (ATCC) as described [61]. In this experiment, PB2, PB1, and PA were all derived from the virus indicated above each panel. Specific site-directed mutations were then introduced and evaluated as indicated in the legend. Polymerase activity was normalized to WT (left side; solid bars) or PB2 265-containing (right side; cross-hatched bars) polymerases at each temperature. Either WT or 265S polymerase activity was arbitrarily set at 100% for ease of interpretation. Data represent mean ± standard deviation (SD) fold increase activity over a no-PB2 control (all transfections were performed in triplicate on three separate occasions). Statistics were performed using one-way ANOVA followed by Tukey’s posttest. * *p* < 0.05, ** *p* <0.01, *** *p* < 0.001.

To investigate whether the PB1 319Q mutation confers temperature sensitivity on polymerases from other influenza A virus strains, we utilized polymerases from two additional strains of IAV: A lab-adapted human isolate, A/Puerto Rico/8/34 H1N1 (PR8), and the pH1N1 pandemic 2009 human isolate, A/California/04/09 pH1N1 (Cal). In both strains, this PB1 mutation synergized with the PB2 265S mutation of LAIV to convey temperature sensitivity (Figure 5). It is notable also that the PB1 319Q mutation conferred a stronger ts phenotype in the genetic background of Cal as compared to PR8 (Figure 5).

### 3.4. PB1 319Q and PB2 265S Impart Temperature Sensitivity to PR8

Having established that the PB1 319Q mutation caused the IAV polymerase to consistently assume a temperature sensitive phenotype, we next aimed to test its effect on viral attenuation in vivo. To do this, we utilized the mouse-adapted strain PR8, which is highly lethal in mice [61]. We rescued PR8-derived viruses with either (i) PB2 N265S (the PB2 mutation of LAIV), (ii) PB1 L319Q, or (iii) both mutations.

**Figure 6:**
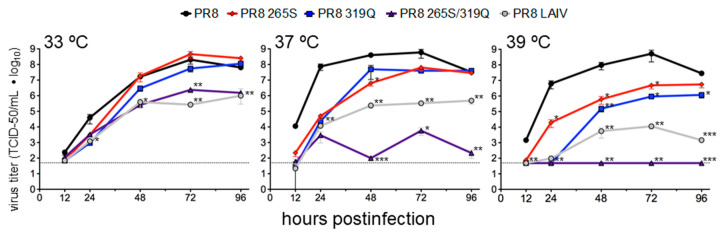
The PB2 265S and PB1 319Q mutations synergistically increased the temperature sensitivity of virus growth in vitro when assessed in the genetic background of PR8. Multicycle growth curve experiments were performed at 33 °C, 37 °C, and 39 °C with A549 cells (ATCC) as described [61]. Mean ± SD values for triplicate infections are plotted. The dotted line denotes the limit of detection (50 TCID_50_/mL). Statistics were performed using one-way ANOVA followed by Tukey’s posttest on log_10_ transformed titers. * *p* < 0.05, ** *p* <0.01, *** *p* < 0.001.

All viruses were sequenced to confirm that the desired mutations were present and that no adventitious mutations were introduced (data not shown). We then analyzed the growth kinetics of these viruses in A549 cells. When the PB2 mutation of LAIV (N265S) was introduced into PR8, the resulting virus displayed temperature sensitivity, with reduced growth at 39 °C in both A549 and MDCK cells (Figure 6). The addition of PB1 L319Q alone resulted in slight temperature sensitivity. However, when PB1 319Q and PB2 265S were both present, virus growth was impaired at temperatures as low as 37 °C (Figure 6). The PR8 265S/319Q double-mutant virus possesses a stronger ts phenotype than a PR8 virus containing all of the LAIV mutations at both 37 °C and 39 °C (Figure 6).

### 3.5. PB1 319Q and PB2 N265S Confer Synergistic Attenuation on PR8 In Vivo

We next sought to examine the attenuation of these viruses in mice. Similar to our previous work with the single mutation at PB1 319 (L to Q), a single mutation at PB2 265 (N to S) attenuated PR8 [61]. Both of these viruses were 10-fold more attenuated than their wild-type counterparts, with an LD_50_ of 300 FFU (Table 3). Note, the LD50 values for the PR8 319Q, PR8 LAIV, and PR8 LAIV with 319Q (Table 3) were taken from our previously published work [55]. The combination of PB2 N265S and PB1 L319Q resulted in an LD50 of 600,000 FFU (Table 3). Consistent with our in vitro findings, this PR8 265S/319Q double mutant virus is 20-times more attenuated than PR8 LAIV (LD_50_ = 30,000 FFU).

### 3.6. The Combination of PB1 319Q and PB2 265S Increases the Formation of Semi-Infectious Particles

In order to understand how the PB1 319Q and PB2 265S mutations might synergize to promote viral temperature sensitivity, we tested whether the polymerase mutations might influence the infectivity of progeny virus in a temperature-dependent manner. To do this, we measured the production of semi-infectious particles (SIPs) using the method of Brookes and Yewdell [62,63]. This approach uses flow cytometry to determine how many infected cells display less than a full complement of influenza proteins at a late timepoint following a single-cycle infection at a low multiplicity of infection.

**Figure 7:**
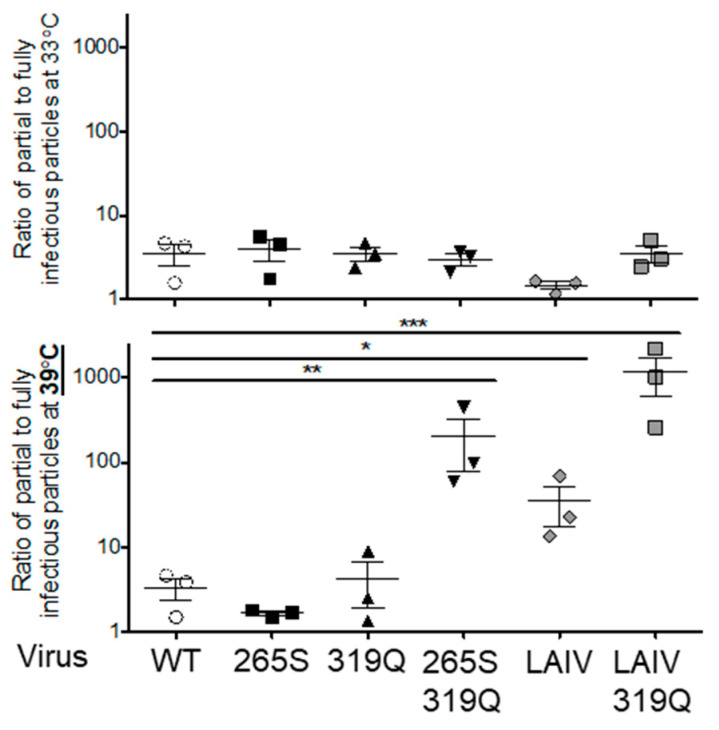
PB2 265S and PB1 319Q synergize to increase the ratio of semi- to fully infectious particles at 39 °C without altering the ratio at 33 °C. MDCK cells were infected at an MOI of 0.01 with viral stocks of either WT, PB1 319Q, PB2 265S, PB1 319Q and PB2 265S, LAIV, and LAIV with PB1 319Q and placed at either 33 °C or 39 °C. After 72 h, viruses were harvested and new cultures of MDCK cells were infected at the permissive temperature of 33 °C with equivalent HA levels of virus from each experimental condition. Neutralizing antibodies to HA were added 2 h post infection to ensure single-cycle infections. At 10 h post infection, cells were lightly trypsinized and were stained for HA and NA. Then, 250,000 cells were run on a flow cytometer to determine the number of single-positive and double-positive cells. The ratio of single-positive cells to double-positive cells is depicted, thus showing the ratio of semi-infectious particles containing only the HA or NA gene segment to fully infectious viruses containing both segments. Experiments were performed in triplicate on separate dates including the treatment of stocks at various temperatures. Statistical analysis: Data were log transformed and analyzed by one-way ANOVA with correction for multiple comparisons using Tukey’s multiple comparison test; * *p* < 0.05, ** *p* < 0.01, *** *p* < 0.001.

We infected MDCK cells at 33 °C and 39 °C with (i) WT, (ii) PB1 319Q, (iii) PB2 265S, (iv) PB1 319Q and PB2 265S, (v) PR8 LAIV, and (vi) PR8 LAIV 319Q viruses. After 72 h, we analyzed the levels of viral particles through HA and infected new plates of cells, with equivalent numbers of viral particles. We ensured a single-cycle infection by introducing an HA-neutralizing antibody (kindly provided by the Yewdell lab) to prevent infection of new target cells. At the permissive temperature of 33 °C, we found that, for each of the viral mutants tested, the majority of virus-infected target cells expressed either HA or NA, but not both proteins (Figure 7). This is consistent with previous findings that the majority of IAV virions fail to express one or more gene segments, and therefore cannot establish a productive infection [62,63]. Unexpectedly, at the elevated temperature of 39 °C, we found that there was a striking difference in the production of SIPs. Specifically, the mutant virus containing both the PB1 319Q and the PB2 265S mutation produced over 20-times more SIPs than any of the single-mutant viruses (or the parental WT virus) (Figure 7). In fact, viruses containing only this pair of mutations had comparable rates of SIP production to PR8 viruses containing either the full complement of LAIV attenuating mutations or the LAIV mutations plus PB1 319Q (Figure 7).

## 4. Discussion

We previously described a novel mutation within the IAV polymerase subunit PB1 at a residue that was conserved across IAV strains [55]. Here, we described the origins/discovery of this mutation and investigated the mechanism by which it is temperature-sensitive. This mutation, leucine to glutamine at residue 319, arose in a virus that contained the PB2 mutation of LAIV (N265S). When introduced into divergent polymerases, this PB1 mutation alone impaired both viral polymerase activity (Figure 5), as measured by minigenome assays, and viral growth (Figure 6). However, the combination of PB1 L319Q with PB2 265S (a mutation contained in LAIV) resulted in a severe temperature-sensitive defect, rendering the polymerase nonfunctional at temperatures as low as 37 °C and dramatically reducing viral growth at 37 and 39 °C without affecting viral replication at 33 °C. When the mouse-adapted strain PR8 was mutated to include either PB1 319Q or PB2 265S, a 10-fold increase in attenuation over the wild-type virus was seen (Table 2). However, the combination of these two mutations resulted in a 20,000-fold increase in attenuation over the wild-type virus (and a 20-fold increase over PR8 containing the full complement of LAIV attenuating mutations).

The exact mechanism of interaction between these two mutations remains to be fully elucidated, but it is notable that the PB1 319Q and PB2 265S double mutant virus exhibited a striking propensity to generate high levels of semi-infectious particles at the nonpermissive temperature of 39 °C. This did not occur at the permissive temperature of 33 °C, and was unique to the double mutant virus (Figure 7). Viruses containing the mutations of LAIV alone also demonstrated this same effect (Figure 7). Consistent with this, Chen et al. previously showed that the attenuating mutations of LAIV altered IAV virion morphology and M1 protein levels in a temperature sensitive manner [38].

A recent editorial by Belshe noted that some methods used to quantitate LAIVs, such as the fluorescent focus assay, “may detect non-infectious vaccine antigens,” thereby leading to underdosing [65] and potentially contributing to the low efficacy of recent H1N1 LAIVs [66,67]. Our data underscore this concern, since we show that LAIVs can generate high levels of semi-infectious particles. At the same time, if this dosing issue were to be corrected, LAIVs that generate SIPs could be advantageous, since they would produce high levels of antigen in the absence of high levels of virus replication. Similarly, the use of LAIVs with increased temperature sensitivity (whose replication would be restricted to the nasal epithelium and upper airway) might permit the safe use of higher doses of vaccine viruses, thereby further increasing their immunogenicity. Additional studies are needed to address these hypotheses.

## 5. Conclusions

The combination of the PB2 265S mutation from the current LAIV and the novel PB1 319Q mutation resulted in a unique IAV phenotype, with greater temperature sensitivity and higher levels of attenuation than the current LAIV in a murine model. This virus also generated increased levels of semi-infectious particles at elevated temperatures. This may result in increased viral attenuation at elevated temperatures, while potentially maintaining near-normal levels of immunogenicity through the production of viral proteins in the absence of fully infectious virus progeny. These findings may have important implications for future efforts to develop improved LAIVs.

## 6. Patents

A.C., S.D., B.K., and J.T. are all inventors on patent 9,878,032 B2 (Attenuated Influenza Vaccines and Uses Thereof), held by the University of Rochester [68].

## Figures and Tables

**Table 1:** Genetic composition of viruses shown in Figure 1.

Virus	PB2	PB1	PA	NP	M	NS	HA	NA
LAIV	A/AA-LAIV	A/AA-LAIV	A/AA-LAIV	A/AA-LAIV	A/AA-LAIV	A/AA-LAIV	A/Korea	A/Korea
SGR	A/AA-LAIV	A/Korea	A/Korea	A/Korea	A/Korea	A/Korea	A/Korea	A/Korea
SGR-rev	A/AA-LAIV	A/Korea	A/Korea	A/Korea	A/Korea	A/Korea	A/Korea	A/Korea

**Table 2:** Amino acid variation between the single gene replacement and revertant viruses. Incidence of residue distribution was determined through the analyze sequence variance function on fludb.org, based on sequencing data current through 2019.

	Amino Acid			Incidence among Human Isolates
		SGR	SGR-Rev	
PB2	73	Q	K	32,794 D 12 K
PB1	145	S	N	145 S 23,711 N
	319	Q	L	0 Q 23,671 L
PA	347	D	N	33,464 D 21 N 14,589 S
	409	S	N	18,919 N
	632	S	P	33,445 S 18 P

**Table 3:** Mutations PB1 319Q and PB2 265S synergize to increase the LD50 of PR8-derived viruses.

Virus	LD_50_^1^
PR8	30 FFU ^2^
PR8 319Q	300 FFU ^2^
PR8 265S	300 FFU
PR8 265S & 319Q	600,000 FFU
PR8 LAIV	30,000 FFU ^2^

^1^ LD50 was determined from survival data of virally infected B6 mice by the method of Reed and Muench [58]. ^2^ LD-50 for PR8 wt, PR8 319Q, and PR8 LAIV are taken from previous work [55].
